# The effect of acute sleep deprivation on skeletal muscle protein synthesis and the hormonal environment

**DOI:** 10.14814/phy2.14660

**Published:** 2021-01-05

**Authors:** Séverine Lamon, Aimee Morabito, Emily Arentson‐Lantz, Olivia Knowles, Grace Elizabeth Vincent, Dominique Condo, Sarah Elizabeth Alexander, Andrew Garnham, Douglas Paddon‐Jones, Brad Aisbett

**Affiliations:** ^1^ Institute for Physical Activity and Nutrition (IPAN) School of Exercise and Nutrition Sciences Deakin University Geelong Australia; ^2^ Department of Nutrition and Metabolism University of Texas Medical Branch Galveston TX USA; ^3^ Appleton Institute Central Queensland University Adelaide Australia; ^4^ Center for Sport Research (CSR) School of Exercise and Nutrition Sciences Deakin University Geelong Australia

**Keywords:** hormones, muscle, muscle protein synthesis, sleep deprivation

## Abstract

Chronic sleep loss is a potent catabolic stressor, increasing the risk of metabolic dysfunction and loss of muscle mass and function. To provide mechanistic insight into these clinical outcomes, we sought to determine if acute sleep deprivation blunts skeletal muscle protein synthesis and promotes a catabolic environment. Healthy young adults (*N* = 13; seven male, six female) were subjected to one night of total sleep deprivation (DEP) and normal sleep (CON) in a randomized cross‐over design. Anabolic and catabolic hormonal profiles were assessed across the following day. Postprandial muscle protein fractional synthesis rate (FSR) was assessed between 13:00 and 15:00 and gene markers of muscle protein degradation were assessed at 13:00. Acute sleep deprivation reduced muscle protein synthesis by 18% (CON: 0.072 ± 0.015% vs. DEP: 0.059 ± 0.014%·h^‐1^, *p* = .040). In addition, sleep deprivation increased plasma cortisol by 21% (*p* = .030) and decreased plasma testosterone by 24% (*p* = .029). No difference was found in the markers of protein degradation. A single night of total sleep deprivation is sufficient to induce anabolic resistance and a procatabolic environment. These acute changes may represent mechanistic precursors driving the metabolic dysfunction and body composition changes associated with chronic sleep deprivation.

## Graphical abstract

Acute sleep deprivation decreases muscle protein synthesis in young, healthy males and females. Acute sleep deprivation promotes a catabolic hormonal environment. Future research should investigate sex‐specific differences.

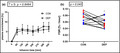

## 1 INTRODUCTION

Acute and chronic sleep loss are linked with a range of negative physiological and psychological outcomes (Kecklund & Axelsson, 2016). While complete sleep deprivation rapidly impedes simple and complex cognitive functions, sleep restriction impairs whole‐body homeostasis, leading to undesirable metabolic consequences in the short‐ and longer‐term (Reutrakul & Van Cauter, 2018). Most metabolic tissues including liver (Shigiyama et al., 2018), adipose tissue (Wilms et al., 2019), and skeletal muscle are at risk of developing sleep loss‐associated adverse outcomes.

Skeletal muscle is a primary regulator of human metabolism. Sleep deprivation (Cedernaes et al., 2015, 2018) and restriction (Harfmann et al., 2015) have the potential to profoundly affect muscle health by altering gene regulation and substrate metabolism. Even relatively short periods of sleep restriction (less than a week) can compromise glucose metabolism, reduce insulin sensitivity, and impair muscle function (Bescos et al., 2018; Buxton et al., 2010). Skeletal muscle is made up of 80% proteins and maintaining optimal muscle protein metabolism is equally critical for muscle health. In situations where skeletal muscle protein synthesis chronically lags protein degradation, a loss of muscle mass is inevitable. Low muscle mass is a hallmark of and precursor to a range of chronic health conditions, including neuromuscular disease, sarcopenia and frailty, obesity, and type II diabetes (Russell, 2010). Population‐based studies report that the risk of developing these conditions is 15%–30% higher in individuals who regularly experience sleep deprivation, sleep restriction, and inverted sleep–wake cycles (Kowall et al., 2016; Lucassen et al., 2017; Wu et al., 2014). To this end, a growing body of evidence suggests that a lack of sleep may directly affect muscle protein metabolism (Aisbett et al., 2017; Monico‐Neto et al., 2013; Saner et al., 2020).

Rodent studies first demonstrated a possible causal link between complete sleep deprivation and disrupted muscle protein metabolism. Rats subjected to 96 hr of paradoxical sleep deprivation, where rapid eye movement sleep is restricted, experienced a decrease in muscle mass (Dattilo et al., 2012) and muscle fiber cross‐sectional area (de Sa et al., 2016). In this model, sleep deprivation negatively impacted the pathways regulating protein synthesis and increased muscle proteolytic activity (de Sa et al., 2016). These findings were paralleled by a human study reporting a catabolic gene signature in skeletal muscle following one night of total sleep deprivation in healthy young males (Cedernaes et al., 2018). To expand on this acute model, investigators recently demonstrated that five consecutive nights of sleep restriction (4 hr per night) reduced myofibrillar protein synthesis in healthy young males when compared to normal sleep patterns (Saner et al., 2020). The possible mechanisms underlying these effects might involve the hormonal environment.

Factors that regulate skeletal muscle protein metabolism at the molecular level are influenced by mechanical (muscle contraction), nutritional (dietary protein intake), and hormonal inputs (Russell, 2010). Testosterone and IGF‐1 positively regulate muscle protein anabolism by promoting muscle protein synthesis (Sheffield‐Moore et al., 1999; Urban et al., 1995), while repressing the genes that activate muscle protein degradation (Zhao et al., 2008). Testosterone binds its specific nuclear receptor, the androgen receptor (AR), at the surface of the muscle fiber and triggers the non‐DNA binding‐dependent activation of the Akt/MTOR pathway (Urban et al., 1995), while IGF‐1 directly upregulates skeletal muscle protein synthesis by activating PI3k/Akt/mTOR (Velloso, 2008). In contrast, cortisol drives catabolism by activating key muscle protein degradation pathways (Kayali et al., 1987). Experimental evidence suggests that acute and chronic sleep loss alter anabolic (Leproult & Van Cauter, 2011; Reynolds et al., 2012) and catabolic (Cedernaes et al., 2018; Dáttilo et al., 2020) hormone secretion patterns in humans. On this basis, we hypothesized that one night of sleep deprivation would decrease muscle protein synthesis and that the hormonal environment may provide a possible mechanism for impaired muscle protein metabolism.

While our understanding of the health consequences of sleep deprivation continues to improve, important gaps and opportunities remain. This includes linking acute mechanistic changes with clinically observable outcomes and moving toward a more prescriptive, individualized understanding of sleep deprivation by examining sex‐based differences. In this study, we sought to determine if one night of complete sleep deprivation promotes a catabolic hormonal environment and compromises postprandial muscle protein synthesis and markers of muscle protein degradation in young, healthy male and female participants.

## 2 METHODS

### 2.1 Participants

Thirteen young (18–35 years old), healthy male and female students gave their informed consent to participate in this randomized, crossover‐designed study. Participants were excluded if they had a history of recent transmeridian travel (i.e., no travel across multiple time zones in the previous 4 weeks), shiftwork (i.e., no involvement in shiftwork over the previous 3 months), frequent napping (i.e., ≥2 naps per week), or had a diagnosed sleep disorder. Participants were required to have habitual bed (22:00–00:00) and wake (06:00–08:00) times that were broadly consistent with the experimental protocol and to self‐report obtaining a minimum of 7 hr of sleep (not time in bed) per night. Chronotype was assessed using the morningness‐eveningness (ME) questionnaire (Horne & Ostberg, 1976). Participants exhibiting extreme morningness (score > 70) or eveningness (score < 30) were excluded. All participants but three displayed an “intermediate” ME type. A detailed account of the strategy for female volunteer recruitment and testing has been comprehensively described by our group (Knowles et al., 2019). Briefly, the effects of female reproductive hormone fluctuations were minimized by testing all female participants during the same phase of their menstrual cycle in both conditions. Although it has previously been shown that the menstrual cycle has no effect on female muscle protein synthesis (Miller et al., 2006), our primary outcome, the follicular phase was avoided to ensure the ratio of estrogen to progesterone was at its lowest. The study was approved by the Deakin University Human Research Ethics Committee (2016‐028) and conducted in accordance with *The Declaration of Helsinki* (1964) and its later amendments. Participants’ physiological characteristics, ME score, and self‐reported habitual time asleep are summarized in Table 1. There was no sex‐specific difference in the ME score (*p* = .148) or self‐reported habitual time asleep (*p* = .401).

**Table 1:** Participants’ characteristics

Sex	Male (*N* = 7)	Female (*N* = 6)
Age	22 ± 1.8	20 ± 1.3
Mass (kg)	71.6 ± 11.3	60.1 ± 10.3*
Height (cm)	173.5 ± 9.0	170.5 ± 5.1
BMI (kg·m^−2^)	22.6 ± 4.1	20.7 ± 3.2
ME score	48.0 ± 6.4	53.8 ± 6.6
Habitual time asleep (self‐reported) (h)	7.5 ± 0.6	7.8 ± 0.7

Note

Mean ± *SD*; BMI, body mass index; ME, morningness‐eveningness score. Significantly different from males, *p < .05.

### 2.2 Sample size calculation

At the time this study was designed, there was no published study investigating the effect of sleep deprivation on muscle protein synthesis. Using data from studies investigating the effects of an anabolic and catabolic stimulus (e.g., immobilization or exercise) on changes in muscle protein synthesis (Lamon et al., 2016; Paddon‐Jones et al., 2006), power analyses conducted on our primary outcome (FSR) indicated that a sample size of 13 would minimize the risk of type II error (*β* = 0.2,*α* = 0.05). Males and females were included as previous work demonstrated that muscle FSR, our primary outcome, is similar in both sexes (Knowles et al., 2019; West et al., 1985).

### 2.3 Prestudy procedure

During the week prior to the study, participants were instructed to maintain their habitual sleep behavior. Participants wore an actigraph (Actical MiniMitter/Respironics, Bend, OR) on their non‐dominant wrist, and completed a sleep diary. The diary was used to corroborate actigraphy data and minimize the possibility of incorrectly scoring periods of sedentary wakefulness as sleep.

Participants completed a control (CON) and experimental (DEP; sleep deprivation) trial in a randomized, crossover design. Trials were separated by at least 4 weeks to allow for a full recovery. Forty‐eight hours prior to each trial, participants were required to refrain from strenuous exercise, alcohol, and caffeine. On the night before (CON) or of the trial (DEP), a standardized meal containing approximately 20% fat, 14% protein, and 66% carbohydrate (energy intake ranging between 8.4 and 8.9 kcal/kg) was provided to participants with water *ad libitum*.

### 2.4 Study procedure

On the night of the sleep deprivation trial (DEP), participants consumed a standardized meal at 19:00 and reported to the laboratory at 21:00 where they were limited to sedentary activities (i.e., reading a book, watching a movie). Participants were constantly observed by research personnel and monitored by actigraphy to ensure they did not fall asleep. They remained in a sound attenuated, light (211 ± 14 lux), and temperature (21 ± 2°C) controlled facility for the entire 30 h protocol. Participants were permitted to consume low‐protein snacks (i.e., fruits and vegetables) and water *ad libitum* during the sleep deprivation period. Regardless of potential differences in insulinemia, adding a non‐pharmacological dose of carbohydrates to a protein synthesis activating dose of proteins (15–30 g) has no additive effect on FSR (Glynn et al., 2013; Hamer et al., 2013; Koopman et al., 2007; Staples et al., 2011), our primary outcome. For the control trial (CON), participants consumed a standardized meal at 19:00 and were permitted to sleep from 22:00 to 07:00 at home, rather than risking a night of disrupted sleep in an unfamiliar laboratory/clinical environment. At 07:00 the following morning, a researcher and nurse with pre‐arranged access to the participants’ home woke the participant and immediately collected a venous blood sample prior to any physical activity or light exposure. Participants were then transported to the laboratory to complete the experimental protocol.

**Figure 1:**
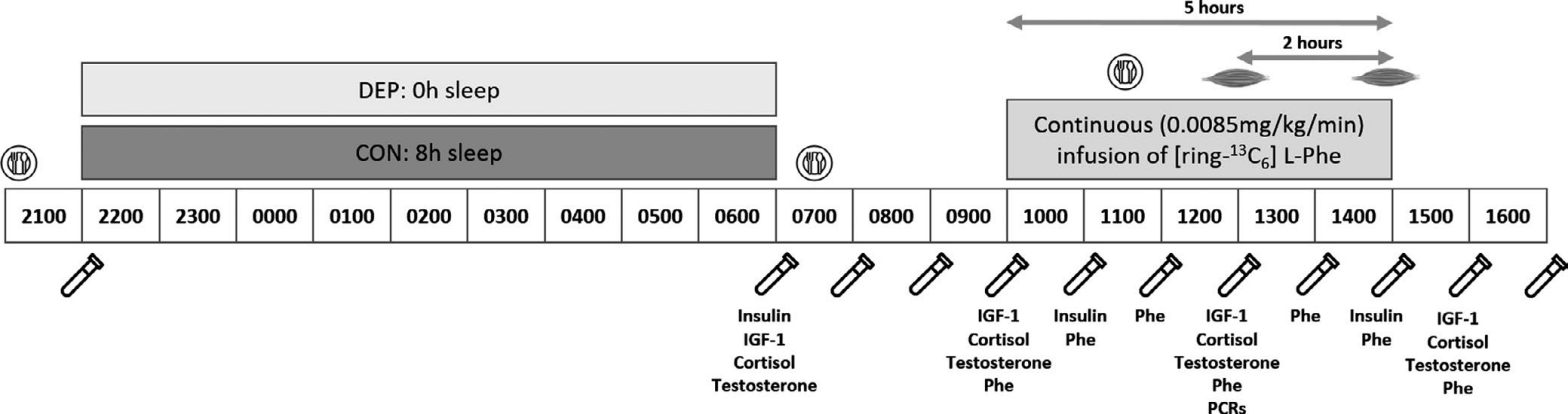
Experimental Protocol. 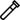 blood collection; 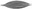: muscle collection; 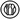: standardized meal. IGF‐1, cortisol, and testosterone concentrations were measured at the 07:00, 10:00, 13:00, and 16:00 timepoints. Insulin concentrations were measured at the 07:00, 11:00, and 15:00 timepoints. Phe enrichment (Phe) was measured in both muscle tissue and blood samples between 10:00 and 16:00. PCRs were run on muscle tissue collected at 13:00

For both DEP and CON trials, at 07:30 participants consumed a standardized breakfast containing approximately 9% fat (7.4 ± 4.2 g), 11% proteins (20.3 ± 1.8 g), and 80% carbohydrates (147 ± 7.2 g). Rather than fasting our participants, standardized meals were provided as part of the experimental protocol for DEP and CON. The goal was to: i) reduce participant discomfort and improve compliance, and ii) model a more realistic and metabolically active, postprandial environment, rather than an overtly catabolic and contrived environment associated with 24 h of fasting. At 08:00, an 18‐gauge cannula was inserted into the antecubital vein of each arm for blood sampling and the primed (0.34 mg·kg^‐1^), constant infusion (0.0085 mg·kg^‐1^·min^‐1^) of L‐[ring‐^13^C_6_]‐ phenylalanine (Cambridge Isotope Laboratories, Tewksbury, MA) from 10:00 to the end of the protocol. At 12:00, participants consumed a standardized lunch containing 12% fat (5.1 ± 1.8 g), 21% protein (20.6 ± 0.3 g) and 67% carbohydrate (65.7 ± 5.6 g). The slowly digested, whole‐food meals reduced the fluctuations in plasma Phe enrichment, thus avoiding the need to add tracer to the meals (Mamerow et al., 2014). Skeletal muscle samples were obtained at 13:00 and 15:00 under local anesthesia (1% Lidocaine) at separate locations from the belly of the *vastus lateralis* muscle using a percutaneous needle biopsy technique as previously described by our group (Lamon et al., 2016). Muscle samples were immediately frozen in liquid nitrogen and used for the measurement of isotopic enrichment and gene expression analysis. An outline of the experimental protocol is presented in Figure 1.

### 2.5 Sleep measures

Sleep was recorded objectively using actigraphy (Actical MiniMitter/Respironics, Bend, OR). The Actical (28 × 27 × 10 mm, 17 g) device uses a piezoelectric omnidirectional accelerometer, which is sensitive to movements in all planes in the range of 0.5–3.0 Hz. Data output from activity monitors (actigraphy) provides an objective, non‐invasive, indirect assessment of sleep and has been validated against polysomnography (Ancoli‐Israel et al., 2003). Primary outcomes were total sleep time and sleep efficiency (total sleep time/time in bed).

### 2.6 Hormone measures

Venous blood samples were collected every hour from 07:00 to 17:00 in EDTA‐tubes, manually inverted, and immediately centrifuged for 15 min at 13,000 rev·min^‐1^ at 4°C. The supernatant (plasma) was then isolated and frozen at −80°C for further analysis. Plasma cortisol, testosterone, and insulin growth factor‐1 (IGF‐1) concentrations were determined using a high‐sensitivity enzyme immunoassay ELISA kit (IBL International, Hamburg, Germany) according to the manufacturer's instructions. Insulin concentration was determined using the MILLIPLEX® MAP Human Metabolic Hormone Magnetic Bead Panel (Merck KGaA, Darmstadt, Germany) according to the manufacturer's instructions.

### 2.7 Isotopic enrichment in plasma

After thawing, plasma was precipitated using an equal volume of 15% sulfosalicylic acid (SSA) solution and centrifuged for 20 min at 13,000 rev·min^‐1^ at 4°C. Blood amino acids were extracted from 500 uL of supernatant by cation exchange chromatography (Dowex AG 50W‐8X, 100–200 mesh H+ form; Bio‐Rad Laboratories). Phenylalanine enrichments were determined by gas chromatography‐mass spectrometry (GC‐MS) using the tert‐butyldimethylsilyl derivative with electron impact ionization as described previously (English et al., 2016). Ions 336 and 342 were monitored.

### 2.8 Isotopic enrichment in muscle proteins

A 30 mg piece of muscle was used for the isolation of mixed muscle‐bound and intracellular protein fractions. Briefly, bound muscle proteins were extracted in perchloric acid and hydrolyzed using 6N hydrochloric acid (110°C for 24 hr). Isotopic enrichments of L‐[ring‐^13^C_6_]‐ phenylalanine in tissue fluid (intracellular fraction) were used as a precursor pool for the calculation of the fractional synthesis rate. Total muscle phenylalanine was isolated using cation exchange chromatography (50W‐8X, 200–400 mesh H+ form; Bio‐Rad Laboratories). Amino acids were eluted in 8 ml of 2N ammonium hydroxide and dried under vacuum. Muscle intracellular and bound protein L‐[ring‐^13^C_6_]‐ phenylalanine enrichments were determined by GC‐MS with electron impact ionization using the tert‐butyldimethylsilyl derivative. Ions 238 and 240 were monitored for bound protein enrichments; ions 336 and 342 were monitored for intracellular enrichments as described previously (English et al., 2016). Mixed muscle protein FSR (%/ hour) was calculated by measuring the direct incorporation of L‐[ring‐^13^C_6_]‐ phenylalanine by the precursor‐product model (Paddon‐Jones et al., 2006):

$$\mathrm{FSR}=\frac{\mathrm{EP}2‐\mathrm{EP}1}{Em\times t}\times 60\times 100$$

where EP1 and EP2 are the bound enrichments of L‐[ring‐^13^C_6_]‐ phenylalanine for the two muscle biopsies, Em is the mean enrichment of L‐[ring‐^13^C_6_]‐ phenylalanine in the muscle intracellular pool, and t is the time interval (min) between biopsies.

### 2.9 RNA extraction and gene expression analysis

Muscle biopsies collected at 13:00 were used for gene expression analysis. RNA was extracted from ~15 mg of skeletal muscle samples using Tri‐Reagent© Solution (Ambion Inc., Austin, TX, USA) according to the manufacturer's protocol. RNA was treated with DNase I Amplification Grade (Thermo Fisher Scientific, MA) and RNA concentration was assessed using the NanoDrop 1,000 Spectrophotometer (Thermo Fisher Scientific). First‐strand cDNA was generated from 1,000 ng RNA using the High Capacity RT‐kit (Applied Biosystems, Carlsbad, CA, USA). cDNA was then treated with RNase H (Thermo Fisher Scientific) according to the manufacturer protocol. Real‐time PCR was carried out using an AriaMx real‐time PCR system (Agilent Technologies, Santa Clara, CA) to measure mRNA levels. mRNA levels for ARNTL (BMAL1), CRY1, PER1, IGF‐1Ea, IGF‐1Eb, FBX032 (atrogin‐1), TRIM63 (MuRF‐1), FOXO1, and FOXO3 were measured using 1 × SYBR© Green PCR MasterMix (Applied Biosystems) and 5 ng of cDNA. All primers were used at a final concentration of 300 nM. Primer details are provided in Table 2. Single‐strand DNA was quantified using the Quant it OliGreen ssDNA Assay Kit (Thermo Fisher Scientific) according to the manufacturer's instruction. ssDNA was used for PCR normalization as previously validated in (Lundby et al., 2005). No differences in ssDNA concentrations were found between groups (data can be found at https://doi.org/10.6084/m9.figshare.12629972.v1). This normalization strategy was cross‐checked against the common housekeeper gene GAPDH (data not shown).

**Table 2:** Primer sequences

Gene	Accession number	Forward	Reverse
ARNTL (BMAL1)	NM_001030272.2	GGCAGCTCCACTGACTACCA	CCCGACGCCGCTTTTCAATC
CRY1	NM_004075.5	CCGTTCCCGGTCCTTTC C	CTAAAGACAAAACGGCCCGC
PER1	NM_002616.3	GAGGACACTCCTGCGACCAG	GCCATGGGGAGAACAGAACA
IGF‐1Ea	NM_001111283.3 and NM_001111284.2	GACATGCCCAAGACCCAGAAGGA	CGGTGGCATGTCACTCTTCACTC
IGF‐1Eb	NM_001111285.3	GCCCCCATCTACCAACAAGAACAC	CAGACTTGCTTCTGTCCCCTCCTTC
FBX032 (Atrogin‐1)	NM_001242463.2	AGTTTCGTGAGCGACCTCAG	CTTTGAAGGCAGGCCGGA
TRIM63 (MuRF‐1)	NM_032588.3	GGGAGGTGATGTCTTCTCTCTG	CTGACAATCGCAGGTCACCC
FOXO1	NM_002015.4	GCAGCCGCCACATTCAACAG	AGAACTTAACTTCGCGGGGC
FOXO3	NM_001455.4	CCGCACGTCTTCAGGTCCTC	CGACGAACATTTCCTCGGCT
GAPDH	XM_006959	CCACCCATGGCAAATTCC	TGGGATTTCCATTGATGACAA

### 2.10 Statistical analysis

Statistical analyses were conducted using SPSS 26.0 (IBM Corp, Armonk, NY). Diagnostic plots of residuals and fitted values were checked to ensure homogeneity of the variance. Hormonal levels were analyzed using a two‐way analysis of variance (ANOVA) with within‐participant factors for time and condition (CON vs. DEP) unless specified otherwise. The Sidak test was used to compare pairs of means when a main effect was identified. For FSR and hormone concentrations, single‐tailed paired *t* tests were used to compare group means. For sleep data and gene expression data, two‐tailed paired *t* tests were used to compare group means. Area under the curve (AUC) was computed on hormone values using the trapezoidal method. The significance levels for the *F* tests in the *t* tests and ANOVA and the Sidak tests were set at *p* < .05. All data are reported as mean ± *SD*.

## 3 RESULTS

### 3.1 Sleep

During the week prior to the study, there were no differences in total sleep time (CON: 5.9 ± 0.5 hr, DEP: 6.1 ± 1.4 hr, *p = *.718) or sleep efficiency (CON: 78.5 ± 6.5%, DEP: 79.4 ± 4.7%, *p = *.801). Similarly, during the night directly preceding the sleep intervention, there were no differences in total sleep time (CON: 6.8 ± 0.8 hr, DEP: 7.4 ± 0.7 hr, *p = *.195) or sleep efficiency (CON: 77.3 ± 6.3%, DEP: 81.0 ± 8.6%, *p = *.424). None of the participants got any sleep during the sleep deprivation intervention or during the testing day (0.0 ± 0.0 hr).

### 3.2 Muscle protein synthesis

**Figure 2:**
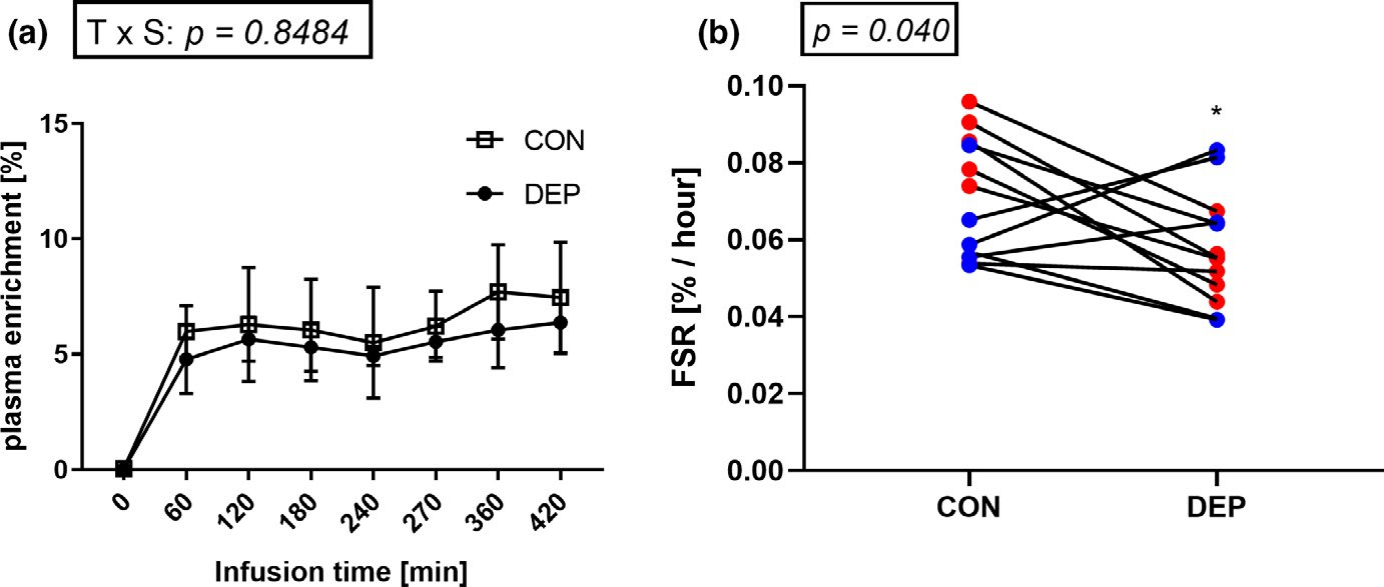
Plasma enrichment of L‐[ring‐^13^C_6_]‐ phenylalanine in a subset of volunteers during the experimental protocol (*N* = 4). Data were analyzed using a two‐way ANOVA. Data are presented as mean ± *SD* (a). Postprandial mixed muscle fractional synthesis rate was measured in the control (CON) and sleep‐deprived (DEP) conditions. Red dots depict male subjects. Blue dots depict female subjects. *N* = 13. Data were analyzed using a single‐tailed paired *t* test. * significantly different from the CON condition, *p < *.05 (b)

Subjects remained in isotopic steady state for the duration of the isotope infusion, with no differences in plasma enrichment between CON and DEP conditions (Figure 2a). Muscle L‐[ring‐^13^C_6_]‐ phenylalanine intracellular enrichments are presented in Table 3. Sleep deprivation reduced postprandial muscle protein fractional synthesis rate (FSR) by 18% (CON: 0.072 ± 0.015% vs. DEP: 0.059 ± 0.014%·h^‐1^, *p = *.040) (Figure 2b). All male, but not female, participants experienced a numerical decrease in protein synthesis in the sleep‐deprived versus control condition.

**Table 3:** Muscle L‐[ring‐^13^C_6_]‐ phenylalanine intracellular enrichments in muscle biopsy samples in the CON or DEP conditions

	CON	DEP
Bx 1	0.045 ± 0.010	0.040 ± 0.011
Bx 2	0.046 ± 0.008	0.042 ± 0.009

Note

Values are means ± SDs tracer‐to‐tracee ratios; *N* = 13. Bx 1–2, muscle biopsy samples 1–2

### 3.3 Plasma testosterone levels

**Figure 3:**
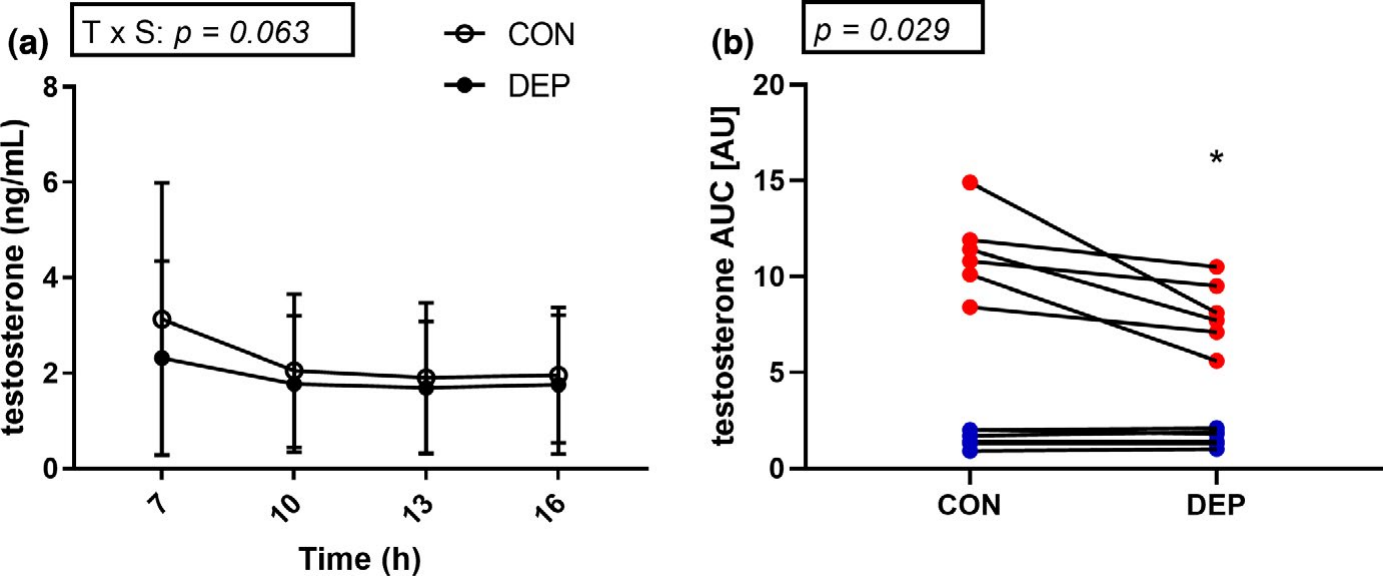
Plasma testosterone concentrations in control (CON) and sleep‐deprived (DEP) conditions. *N* = 13. Data were analyzed using a two‐way ANOVA. Data are presented as mean ± *SD* (a). Area under the curve calculated for plasma cortisol concentrations. Red dots depict male subjects. Blue dots depict female subjects. *N* = 13. Data were analyzed using a single‐tailed paired *t* test *; significantly different from the CON condition, *p < *.05 (b)

There was a main effect of time (*p = *.002) but the interaction effect of sleep × time for plasma testosterone levels did not reach statistical significance (*p = *.063; Figure 3a). The area under the curve decreased by 24% in the DEP condition (CON: 6.40 ± 5.28 AU vs. DEP: 4.86 ± 3.64 AU, *p = *.029; Figure 3b). A male and a female sub‐population group were visually observed, where all male subjects had their testosterone AUC decreasing in the DEP condition (Figure 3b).

### 3.4 Plasma cortisol levels

**Figure 4:**
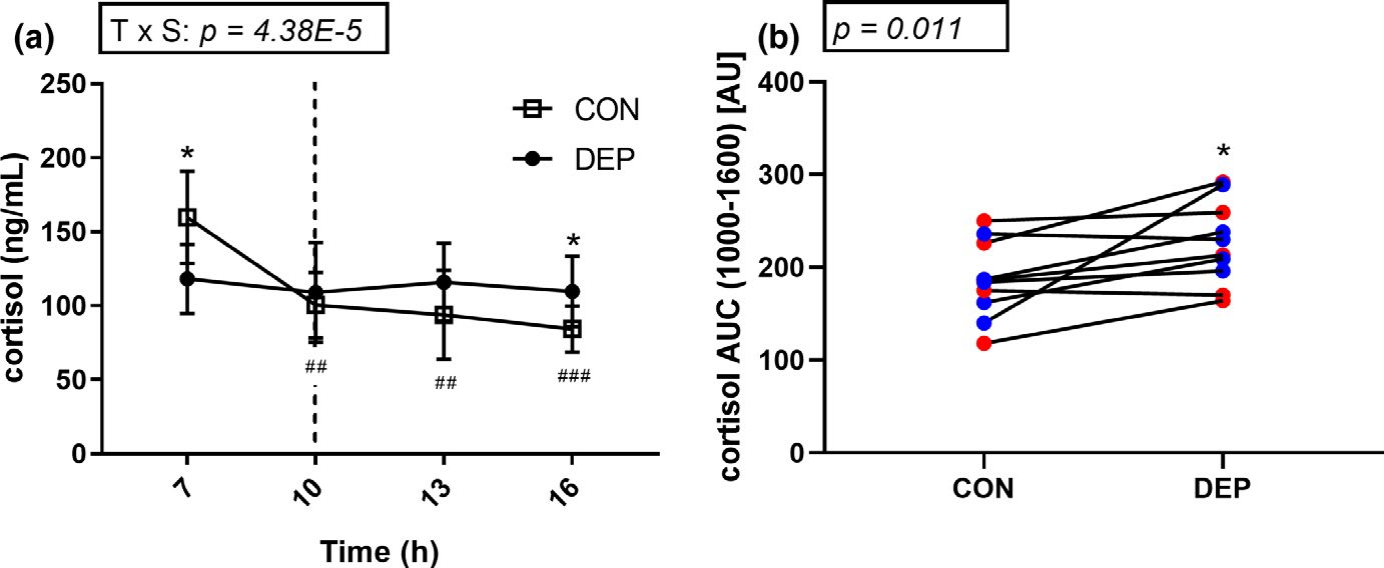
Plasma cortisol concentrations in control (CON) and sleep‐deprived (DEP) conditions. *N* = 13. Data were analyzed using a two‐way ANOVA. * significantly different from the CON condition, *p < *.05. ^##^; CON group was significantly different from the 07:00 timepoint in the CON group, *p* < .01. ^###^; CON group was significantly different from the 07:00 timepoint in the CON group, *p* < .001. Data are presented as mean ± *SD* (a). Area under the curve calculated for plasma cortisol concentrations from 10:00 (dashed line, a) until the end of the protocol. Red dots depict male subjects. Blue dots depict female subjects. *N* = 13. Data were analyzed using a single‐tailed paired *t* test *; significantly different from the CON condition, *p* < .05. (b)

A significant interaction effect of sleep × time (*p* = 4.38E‐5) was observed for plasma cortisol levels. Consistent with the typical increase in cortisol observed during the later stages of sleep (Vargas & Lopez‐Duran, 2020), plasma cortisol levels were significantly higher (*p* = .014) in the CON condition than in the DEP condition at 07:00 (wake time for the control condition). At 10:00, plasma cortisol was similar in both sleep conditions (*p* = .940), but by 16:00, cortisol was significantly higher in the DEP condition (*p* = .048) (Figure 4a). Plasma cortisol area under the curve (10:00–16:00), was 21% higher during DEP than CON (CON: 186 ± 41.7 AU vs. DEP: 226 ± 44.6 AU, *p* = .011) (Figure 4b).

### 3.5 Insulin and IGF‐1 levels

**Figure 5:**
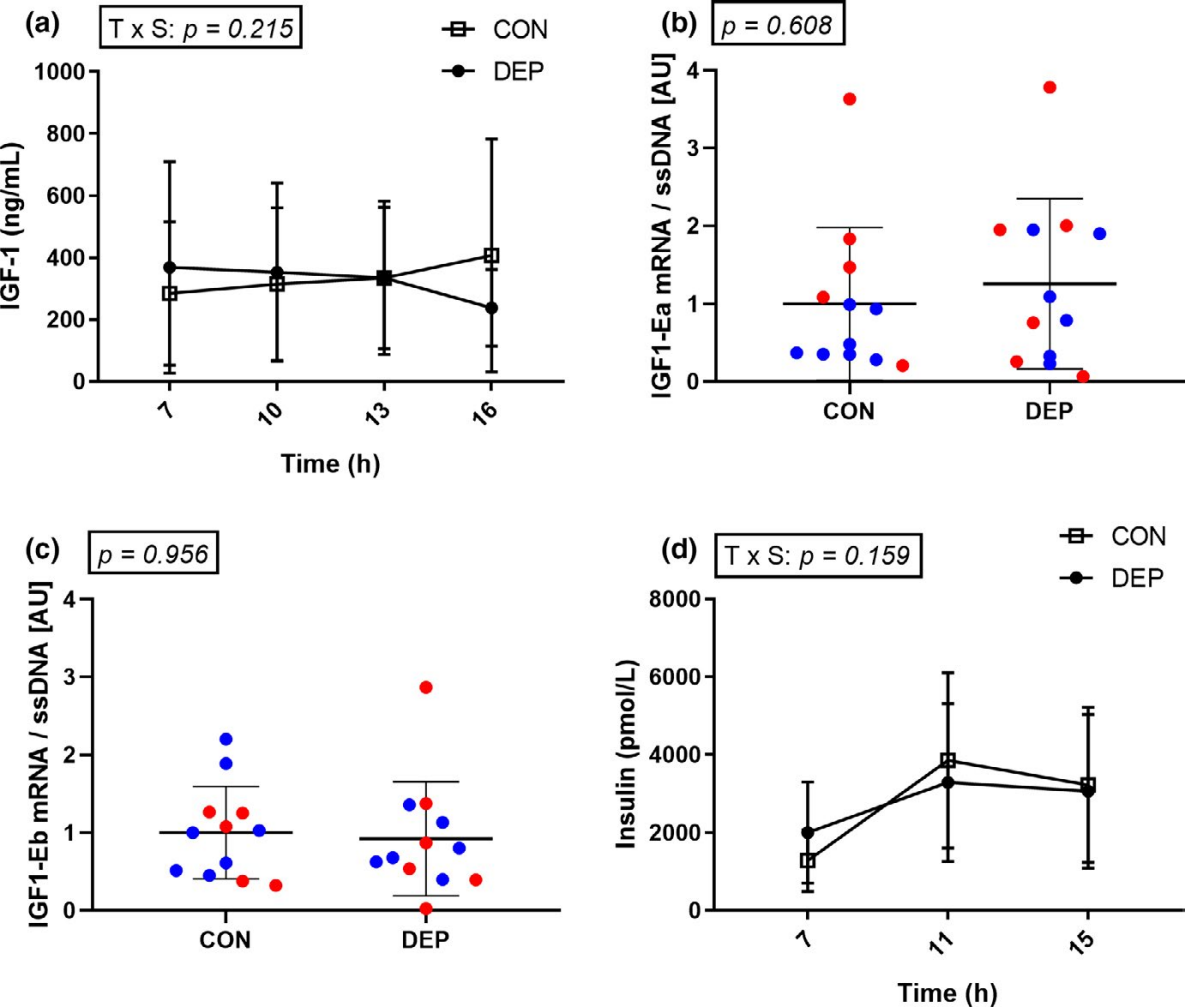
Plasma IGF‐1 concentrations in control (CON) and sleep‐deprived (DEP) conditions. *N* = 13. Data were analyzed using a two‐way ANOVA (a). Muscle mRNA levels of the IGF‐1 isoforms IGF1‐Ea (b) and IGF1‐Eb (c) in muscle biopsies collected at 13:00. Red dots depict male subjects. Blue dots depict female subjects. *N* = 13. Data were analyzed using a two‐tailed paired *t* test. Insulin concentrations in control (CON) and sleep‐deprived (DEP) conditions. *N* = 13. Data were analyzed using a two‐way ANOVA (d). All data are presented as mean ± *SD*

Plasma IGF‐1 concentrations did not vary with time, sleep, or the combination of both (Figure 5a). Similarly, sleep deprivation did not influence the muscle expression levels of IGF1 mRNA isoforms IGF1‐Ea and IGF1‐Eb when measured in the postprandial state (Figure 5b,c). Plasma insulin concentrations varied across the day, but there was no effect of sleep or the combination of sleep and time (Figure 5d).

### 3.6 Gene expression

**Figure 6:**
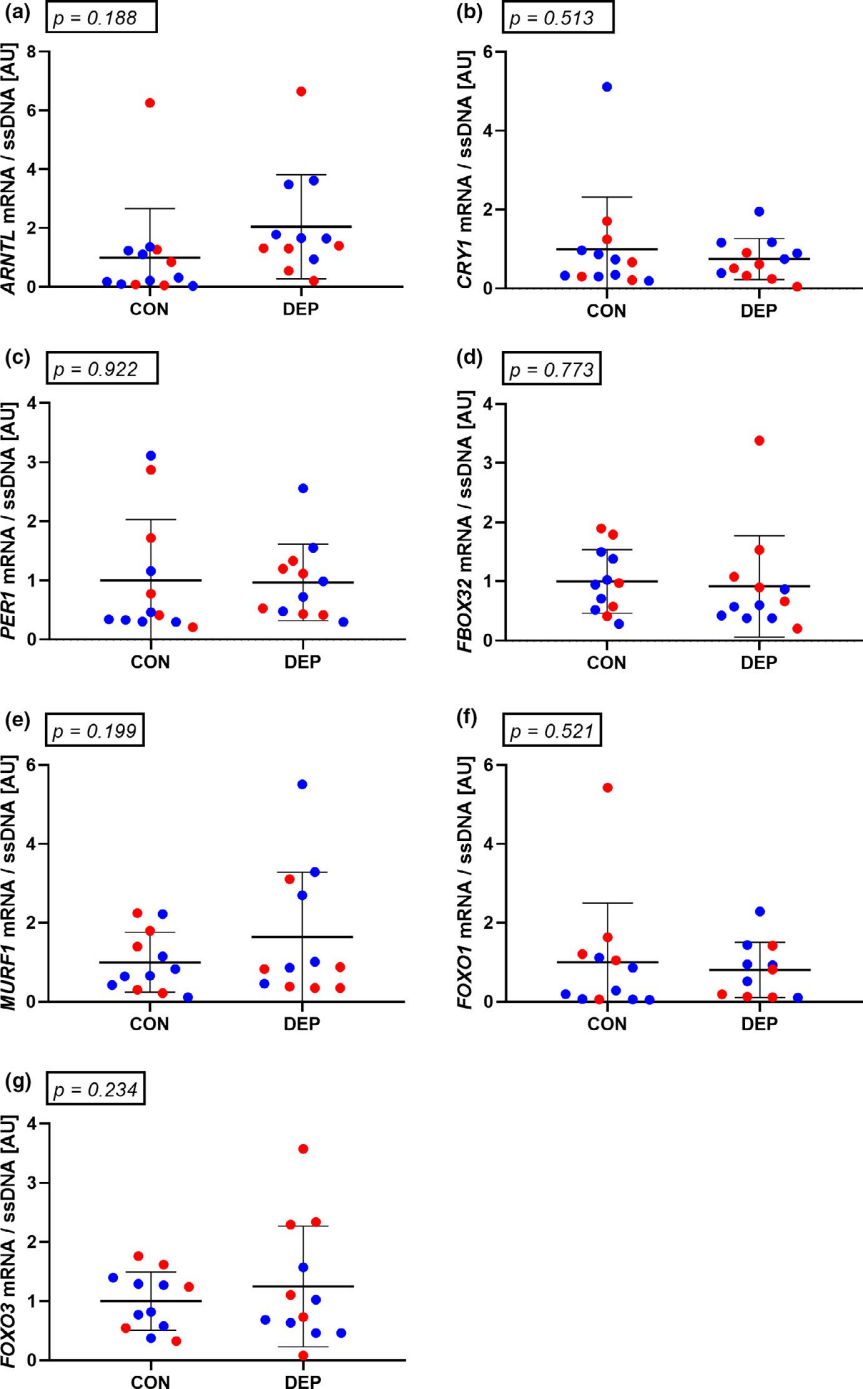
Muscle mRNA levels of *ARNTL* (a), *CRY1* (b), *PER1* (c), atrogin (*FBOX32*) (d), *MURF1* (e), *FOXO1* (f), and *FOXO3* (g) in muscle biopsies collected at 13:00. Red dots depict male subjects. Blue dots depict female subjects. *N* = 13. Data were analyzed using a two‐tailed paired *t* test. All data are presented as mean ± *SD*

The muscle expression levels of core clock genes *ARNTL, CRY1,* and *PER1* or muscle protein degradation markers *FBOX‐32, MURF1, FOXO1,* and *FOXO3* were assessed in muscle biopsies collected in the postprandial state and did not change in response to sleep deprivation (Figure 6a‐g).

## 4 DISCUSSION

Chronic sleep loss is a potent catabolic stressor (Cedernaes et al., 2018; Saner et al., 2020) that increases the risk of metabolic dysfunction (Reutrakul & Van Cauter, 2018) and is associated with a loss of muscle mass and function at the population level (Lucassen et al., 2017; Piovezan et al., 2015). In this study, we have demonstrated that a single night of sleep deprivation is sufficient to induce anabolic resistance, reducing postprandial skeletal muscle protein synthesis rates by 18%. This decrease was accompanied by an acute, pro‐catabolic increase in plasma cortisol and a sex‐specific reduction in plasma testosterone. Our study is the first to demonstrate that acute sleep deprivation blunts muscle protein synthesis, a key regulator of skeletal muscle turnover. It adds to early results reporting a reduction in muscle protein synthesis following five nights of sleep restriction (Saner et al., 2020) and provides insights into the mechanisms underlying the suppression of anabolism following an acute or chronic lack of sleep.

### 4.1 Acute sleep deprivation decreases muscle protein synthesis

One night of sleep deprivation significantly reduced postprandial skeletal muscle protein synthesis in a population of healthy young adults. In rodents, complete sleep deprivation is known to decrease muscle mass (Dattilo et al., 2012), muscle fiber cross‐sectional area (de Sa et al., 2016), and markers of the protein synthesis pathways. Only one study to date has investigated the effect of poor sleep on muscle protein synthesis in humans. Using a chronic sleep restriction model, Saner *et al*. recently reported that five consecutive nights of sleep restriction reduced muscle protein synthesis rates in healthy young males (Saner et al., 2020). Despite employing a different stable isotope (phenylalanine vs. deuterated water), study design (acute cross‐over design vs. chronic parallel design), and population (males and females vs. males only), our findings support those by Saner et al. Negative phenotypic outcomes associated with a period of chronic sleep deprivation likely reflect a metabolic shift toward catabolism due to the accumulation of blunted anabolic responses to protein‐containing meals and physical activity. Our group has further discussed the results and implications of the Saner paper elsewhere (Knowles, 2020).

A novel, exploratory outcome from our study highlights the potential for sex‐specific responses to sleep deprivation. While the study was not powered to formally compare sexes, all of our male, but not female, participants experienced a numerical decrease in protein synthesis in the sleep‐deprived versus control condition. Since we could not identify individual characteristics or behaviors consistent with the paradoxical increase in muscle protein synthesis observed in some of our female participants, our data may reflect a broader, sex‐specific physiological response and warrants more focused attention.

To balance experimental rigor with subject comfort and improve the potential clinical translation of our data during both sleep trials, participants consumed a standardized meal prior to the first muscle biopsy. Dietary protein is a potent activator of muscle protein synthesis, potentially explaining the slightly higher protein synthesis rates we observed, compared to typical data obtained from fasted participants (Kumar et al., 2009). By studying participants in the postprandial state, we were able to conclude that acute sleep deprivation induces anabolic resistance, decreasing the capacity of the muscle to respond to the typical anabolic stimulation triggered by dietary protein intake. These results have potential, far‐reaching implications for the musculoskeletal and metabolic health of populations including shiftworkers, new parents, students, and older adults, who are at increased risk of acute and/or chronic sleep loss. Future research and clinical interventions prioritizing nutrition and/or protein‐synthesis stimulating exercise (Saner et al., 2020) is warranted as these may represent practical and effective means of protecting muscle mass and function in sleep‐deprived populations.

### 4.2 Acute sleep deprivation promotes a less anabolic hormonal environment

Testosterone AUC was reduced by 24% following one night of sleep deprivation. There is limited evidence describing how complete sleep deprivation alters testosterone daytime secretion patterns. In males, plasma testosterone fluctuates during the day, with concentrations increasing during sleep and gradually decreasing during waking periods (Axelsson et al., 2005; Touitou et al., 1990), with marginal circadian effects (Axelsson et al., 2005). A minimum of 3 hr of normal sleep, including paradoxical sleep opportunities (Luboshitzky et al., 2001), is required to increase testosterone. In line with our findings, an earlier study in healthy young males found that one night of acute sleep deprivation did not alter 24 hr testosterone AUC; however, a pattern similar to ours could be observed across the day (Dáttilo et al., 2020).

In males, testosterone is a potent regulator of muscle protein synthesis both on the short (5 days) (Sheffield‐Moore et al., 1999) and longer term (4 weeks) (Urban et al., 1995). However, acute exposure to testosterone is not sufficient to alter postabsorptive muscle protein synthesis or degradation rates over a 5‐hr period (Church et al., 2019). Whether transiently low testosterone levels can negatively impact muscle protein synthesis rates in the fasted and/or fed state is unknown and constitutes a challenge to validate experimentally. However, others have shown that testosterone secretion is depressed during sleep deprivation (Luboshitzky et al., 2001), and we and others (Dáttilo et al., 2020) have observed another low testosterone secretion period during the daytime, primarily prior to our tracer infusion period. Whether this phasic response may contribute to anabolic resistance needs to be tested experimentally.

Finally, testosterone is the major androgenic hormone, but is also present in females, albeit in concentrations that are 10‐fold lower than typical male levels (Vingren et al., 2010). A sex‐specific pattern, which was not tested statistically due to sample size limitations, could be visually observed in our study. The negative effect of sleep deprivation on testosterone levels appeared particularly pronounced or inherent to males. Whether this difference may be causative of potential differences in the protein synthesis results cannot be addressed by our study and warrants further sex‐specific investigations.

### 4.3 Acute sleep deprivation promotes a more catabolic hormonal environment but no difference in gene expression

Consistent with previous studies conducted in males, a cortisol response upon awakening was not observed following one night of acute sleep deprivation (Vargas & Lopez‐Duran, 2020). This blunted cortisol response was accompanied by a chronically higher cortisol secretion across the day when calculated without accounting for the cortisol awakening response (Cedernaes et al., 2018; Dáttilo et al., 2020). While our results are in line with these observations, the participants’ night‐time calorie consumption needs to be acknowledged as a potential confounder. Previous studies have shown that over‐night glucose infusion reduces cortisol levels (Benedict et al., 2009). In our study, cortisol AUC was, however, calculated after the 10:00 time point, after nutrient and energy intake were normalized. Further, post hoc analyses revealed that, while the control group displayed a gradual, significant decrease in cortisol across the day, no differences were observed in the sleep‐deprived group at any time point, indicating a potential circadian misalignment. Cortisol has catabolic properties. In rats, corticosterone reduced muscle protein synthesis, while increasing myofibrillar protein breakdown (Kayali et al., 1987). In contrast, in humans, acute hypercortisolemia did not affect muscle fractional synthesis rates but blunted the net muscle protein balance (Paddon‐Jones et al., 2003), suggesting that cortisol preferentially increases muscle protein breakdown, rather than blunting muscle protein synthesis. Indeed, complete sleep deprivation can lead to a catabolic gene signature in human skeletal muscle (Cedernaes et al., 2018), which might be reflective of an increase in muscle protein degradation. In our acute model, we, however, failed to observe any difference in the muscle expression levels of the proteolytic genes *FOXO1* and *FOXO3*, or in the expression levels of muscle‐specific atrogenes Atrogin‐1 (*FBXO32*) and *MURF1*. This may be explained by the fact that our muscle biopsy was collected at a later time point (07:30 vs. 13:00 in our study), but also by the postprandial state of our participants at the time of sample collection. Indeed, the consumption of a mixed meal attenuates ubiquitin‐mediated proteolysis when compared to fasted (Carbone et al., 2013). Whilst it should be kept in mind that acute studies essentially report “snapshots” of chronic processes, our choice to use a postprandial model has the advantage of providing a better reflection of time periods where the anabolic flux is greater. Supporting an effect of poor sleep on muscle protein degradation, some authors also recently suggested that poor sleep‐induced hypercortisolemia might play a role in the development of sarcopenia (Piovezan et al., 2015), with potential sex‐specific effects (Buchmann et al., 2016); however, further research is required to establish cause‐and‐effect relationships.

In contrast to others (Cedernaes et al., 2015), we did not observe a decrease in the muscle expression levels of the core clock genes following a night of total sleep deprivation. Since the timing of muscle sample collection was different, it can be hypothesized that the muscle circadian rhythm might have been able to realign over this time period. It should also be acknowledged that night time calorie consumption constitutes a potential confounder as food intake can act as a “Zeitgeber” for peripheral tissues in mammals (Challet, 2019), including skeletal muscle. However, differences in core clock gene expression might not be reflective of a physiologically significant change. This warrants the comparison of more functional readouts, such as protein expression levels, which was not possible in this study due to tissue availability.

### 4.4 Strengths and limitations

For the same reason, protein expression levels of the molecular regulators of muscle protein synthesis could not be assessed. To improve compliance, comfort, and retention, we were requested by our human ethics committee to allow participants to consume low‐protein snacks (i.e., fruits and vegetables), and water *ad libitum* before normalizing calorie consumption at the 07:00time point, 6 hours before the first biopsy was collected. This strategy was effective in achieving similar plasma insulin levels across the two conditions at all time points. While in line with the existing literature, our results should, however, be considered in the light of the potential influence of carbohydrate ingestion on plasma hormone concentrations, and future research should look at suppressing or normalizing overnight glucose intake. Using the home environment constituted a compromise as it avoids the need for habituation, which is a strength of this study, but rules out the ability to obtain overnight blood samples. Despite the presumed familiarity with their home sleep environment, our participants were mildly sleep‐restricted in the week coming into both arms of the study, though total sleep times still fell within the stated sleep‐wake time inclusion criterion. Sleep recorded on the pre‐trial nights approximated their self‐reported 7‐hr typical duration. Whether the mild‐sleep restriction state had any impact on the results remains unclear. Future studies may consider the inclusion of laboratory sleep trials and gold‐standard polysomnographic sleep measures permitting analyses of sleep quality. While increasing participant burden, this would allow the characterization of relationships between measures of sleep quality and physiological outcomes, including skeletal muscle protein synthesis and the hormonal environment.

In conclusion, poor quantity and quality sleep is linked to a range of metabolic outcomes. Our study demonstrates that total sleep deprivation induces anabolic resistance by reducing postprandial muscle protein synthesis in young males and females. Sleep deprivation also promoted a catabolic environment, providing insights into the possible mechanisms underlying this process. In designing this clinical trial, we did, however, not focus on sex‐specific differences, nor did we power our study in order to detect such differences. Therefore, the potential sex‐based differences observed in this study are not conclusive but prompt a dedicated investigation to better examine links between inadequate sleep and impaired skeletal muscle health in male and female cohorts.

## CONFLICT OF INTEREST

The authors have no conflict of interest to declare.

## AUTHOR CONTRIBUTIONS

SL, GEV, and BA designed the study. SL, AM, OK, DC, SEA, AG collected the data. SL, EAL, OK, GEV, SEA, DPJ processed and analyzed the data. SL conducted statistical analyses and drafted the manuscript. SL and BA supervised the project. All authors commented on and edited the manuscript drafts.
